# Molecular biomarkers in multiple sclerosis

**DOI:** 10.1186/s12974-019-1674-2

**Published:** 2019-12-23

**Authors:** Tjalf Ziemssen, Katja Akgün, Wolfgang Brück

**Affiliations:** 1 MS center, Center of Clinical Neuroscience, University Clinic Carl-Gustav Carus, Dresden University of Technology, Dresden, Germany; 2 Institute of Neuropathology, University Medical Center, Göttingen, Germany

**Keywords:** Multiple sclerosis, Molecular biomarker, Oligoclonal bands, Neurofilament, Cerebrospinal fluid, Treatment response

## Abstract

Multiple sclerosis (MS) is an inflammatory-neurodegenerative disease of the central nervous system presenting with significant inter- and intraindividual heterogeneity. However, the application of clinical and imaging biomarkers is currently not able to allow individual characterization and prediction. Complementary, molecular biomarkers which are easily quantifiable come from the areas of immunology and neurobiology due to the causal pathomechanisms and can excellently complement other disease characteristics. Only a few molecular biomarkers have so far been routinely used in clinical practice as their validation and transfer take a long time. This review describes the characteristics that an ideal MS biomarker should have and the challenges of establishing new biomarkers. In addition, clinically relevant and promising biomarkers from the blood and cerebrospinal fluid are presented which are useful for MS diagnosis and prognosis as well as for the assessment of therapy response and side effects.

## Introduction

Multiple sclerosis (MS) is a chronic autoimmune disease characterized by inflammatory demyelination and neurodegeneration in the central nervous system (CNS) [1]. The disease shows a great heterogeneity with regard to radiological and histopathological changes, clinical appearance and progression, as well as therapy response [2–6]. It is therefore very important to define specific features of the disease that facilitate diagnosis and prognosis and allow an assessment of the therapeutic response and risk of side effects [7–9]. Currently, the lesion load in the CNS determined by magnetic resonance imaging (MRI) as well as clinical characteristics, e.g., relapse rate and disability progression, play the most important role [10]. However, although it is possible to quantify and standardize these characteristics in larger patient groups, it is not possible until now in individual patients [11, 12].

Molecular biomarkers, on the other hand, are easily quantifiable and can excellently complement MRI and clinical characteristics [13]. Biomarkers for MS come from the areas of immunology and neurobiology due to the causal pathomechanisms [14]. Although the importance of molecular biomarkers has been increasingly recognized in recent years, their validation is a lengthy process, so that only a few biomarkers have so far been routinely used in clinical practice [15]. However, the number of potential biomarkers at different stages of testing is promising. This review describes the characteristics that an ideal MS biomarker should have and the challenges of establishing new biomarkers [16]. In addition, clinically relevant and promising biomarkers from the blood and cerebrospinal fluid (CSF) are presented which are useful for MS diagnosis and prognosis as well as for the assessment of therapy response and side effects.

## What makes an ideal MS biomarker?

A biomarker is defined as a characteristic that can be objectively measured and evaluated and serves as an indicator of normal biological processes, pathological processes or pharmacological reactions to therapy [17]. Ideally, this is a binary system, in other words a characteristic that is present in people with a certain disease and is absent in healthy people or people with another disease or vice versa. If the disease worsens or improves, the concentration of the biomarker should increase or decrease accordingly [18]. Another characteristic of an ideal biomarker is that it is safe for the patient and as easy to detect as possible, in the best case it is a non-invasive method. The analytical detection method should be highly accurate and reproducible and at the same time fast, simple, and cost-effective in order to ensure comprehensive implementation [19]. Thereby, the result of the detection method should be insensitive to systematic influencing factors such as sample collection, sample processing, and sample storage [20].

In addition to the typical clinical characteristics of a disease, imaging biomarkers are often used with the aid of imaging methods. In MS, for example, MRI provides information on the size, number, age, and development of lesions in the CNS and plays an important role in diagnosis and therapy monitoring [21–23]. In the future, brain atrophy could also gain importance if its measurement becomes possible in individual patients [24–29]. Imaging biomarkers are distinguished from molecular biomarkers, which comprise deoxyribonucleic acid (DNA), ribonucleic acid (RNA), and proteins. The advantages of DNA as a molecular marker are a less demanding handling as well as an easier and less expensive detection [30]. In contrast, RNA and proteins are quantitative characteristics that, as opposed to DNA, are suitable for monitoring disease specific processes. In the field of MS, all established molecular biomarkers are currently proteins, most of them antibodies [15, 31, 32].

For the detection of molecular biomarkers, a sample must be taken from the patient. In MS, the body fluids blood and CSF, which offer different advantages and disadvantages, are particularly suitable (Table 1). Since blood collection is the less invasive procedure, the validation of new molecular biomarkers should examine whether serum or plasma detection is as suitable as CSF detection.

**Table 1:** Advantages and disadvantages of the detection of biomarkers in blood and cerebrospinal fluid; modified according to [33, 34]

	Advantages	Disadvantages
Blood	• Safe, quick, and easy collection • Different timepoints could be measured • Quite large quantities can be analyzed	• Does not necessarily reflect changes in the CNS • Diurnal variation of many soluble markers • Markers affected by a lot of processes (degradation, concomitant disease…) • Potential preanalytical bias • Lower concentration of potential biomarker
Cerebrospinal fluid	• Best reflects the processes in the CNS • Lower concentration of potential biomarker	• Exposure to invasive lumbar puncture • Only low quantities can be obtained • Difficult to measure different timepoints • Potential preanalytical bias

## Challenges in the establishment of biomarkers

In the development and establishment of new biomarkers, the above-mentioned properties of an ideal biomarker have to be taken into account and some additional difficulties have to be overcome, which are described below [35].

Sensitivity and specificity are two key figures of biomarkers. Sensitivity describes the proportion of true positive test results among those who are actually affected by the disease. Specificity, on the other hand, indicates the proportion of true negative results among those who are not ill (Table 2) [36]. Since high sensitivity is usually at the expense of specificity and vice versa, it is of great importance to identify biomarkers that achieve a satisfactory balance of both properties. Other important key figures are the positive and negative predictive value of a biomarker. These indicate the proportion of patients with a positive or negative test result who are correctly diagnosed.

**Table 2:**
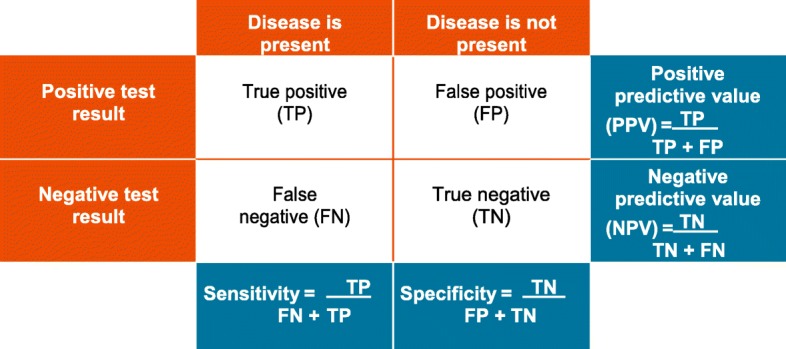
Representation of the sensitivity and specificity as well as the positive and negative predictive value of a biomarker using the example of diseased and non-diseased test subjects

Various analytical methods are often available for the detection of molecular biomarkers. However, the use of different detection methods can lead to differing test results and thus severely limit the informative value of the biomarker. Research on interleukin (IL)-21 as potential biomarker to predict the risk of secondary autoimmunity after alemtuzumab therapy shows that even the exchange of individual components using the same detection method can change the results [37]. In this case, the use of other ELISA (enzyme-linked immunosorbent assay) kits for determining the concentration of IL-21 in the serum no longer showed a predictive association [38]. Therefore, the development of molecular biomarkers requires validation with different detection methods [39].

Initial investigations of new biomarkers usually take place in small patient groups, followed by confirmation of the biomarker candidates in large, independent cohorts. However, this transfer of the results to large populations is not always successful. For example, anti-myelin oligodendrocyte glycoprotein (MOG) and anti-myelin basic protein (MBP) antibodies were identified in a study of 103 patients as predictors for the development of MS after a first demyelinating event [40]. In a subsequent study with 462 participants, however, this could not be confirmed [41]. The same was observed for the anti-KIR4.1 antibody [42] which had previously been proposed as a biomarker for MS diagnosis [43]. Biomarker development is comparable with drug development as independent validation has to be demonstrated in large cohorts after positive pilot test. If biomarker tests are going to be used to drive patient care, than an understanding and careful assessment of these concepts are essential, since “A Bad Biomarker Test Is as Bad as a Bad Drug” [44].

**Fig. 1:**
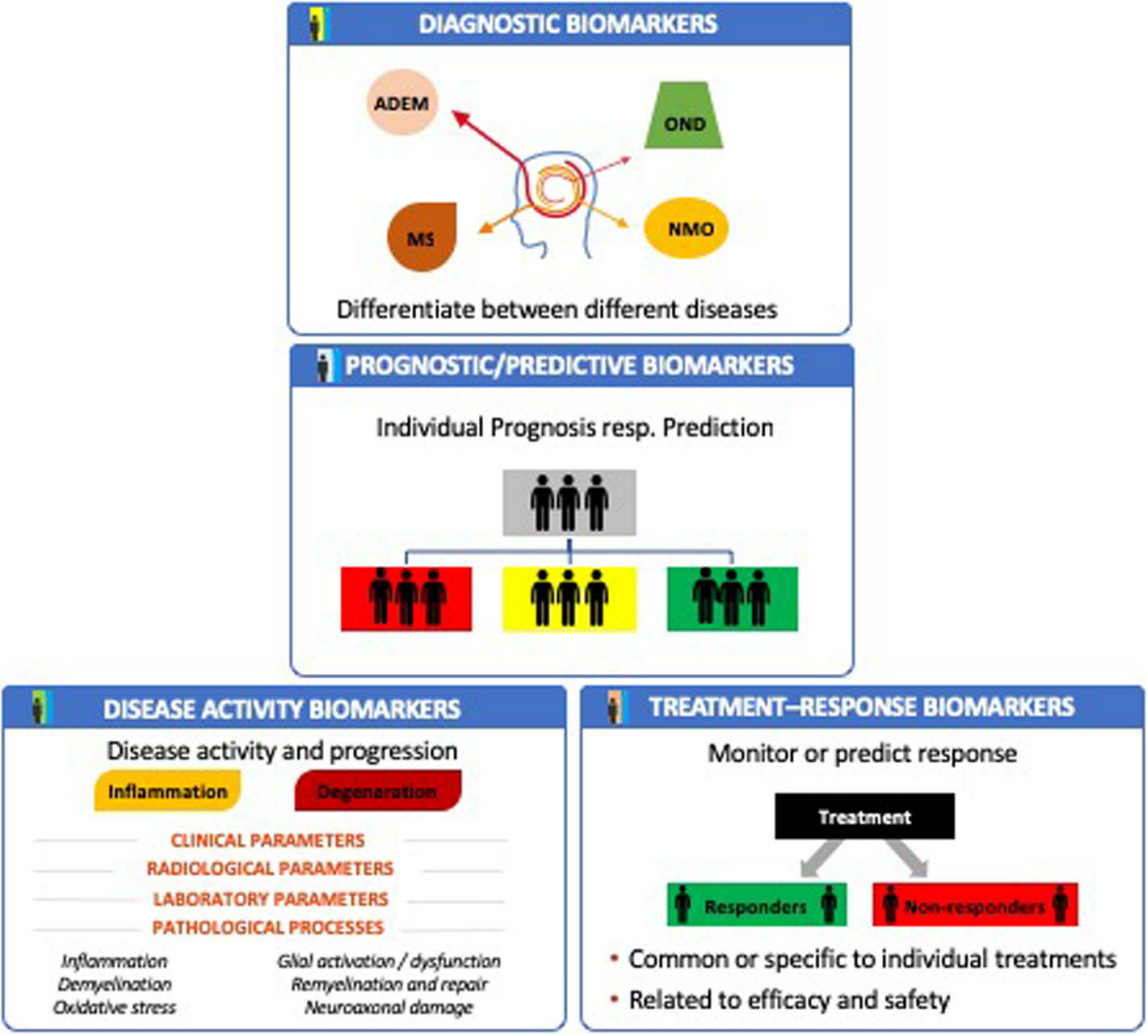
Different types of biomarkers in multiple sclerosis: **Diagnostic biomarkers** are used to confirm the diagnosis of MS. A test used to diagnose a disease often measures a type of biomarker called a “surrogate.” Diagnostic biomarkers may facilitate earlier detection of a disorder than can be achieved by other approaches. A **prognostic biomarker** helps to indicate how a disease may develop in an individual when a disorder is already diagnosed. The presence or absence of a prognostic marker can be useful for the selection of patients for treatment but does not directly predict the response to a treatment. This is more specified by the **predictive biomarker** which helps to determine which patients are most likely to benefit from a specific treatment option. Predictive diagnostics can provide information about how well a treatment is likely to work in a particular patient or about the likelihood of that treatment causing an unwanted side effect. For prognosis and prediction, **disease activity biomarkers** comprise biomarkers to measure inflammatory and/or neurodegenerative components of disease. For personalized MS treatment, **treatment-response biomarkers** could be helpful to differentiate patients regarding their outcome related to efficacy and side effects (treatment responders and non-responders as well as patients with and without adverse drug reactions). In addition, these treatment-response markers could be applicable for all treatments or be specific for a specific treatment only

Due to the challenges described above in establishing new biomarkers, careful validation of potential candidates is essential [45]. In doing so, the robustness of the detection procedure should be checked and the validity of the results in large patient populations confirmed. As a result, the validation process is often lengthy and usually takes between 5 and 15 years [46, 47]. Therefore, the expansion of the repertoire of biomarkers for MS has so far been slow. Molecular biomarkers can complement MRI and clinical markers in different phases of MS disease. These include diagnosis and prognosis as well as response to progression-modifying therapies and the occurrence of side effects. The different types of biomarkers in MS are presented in Fig. 1.

## Molecular biomarkers for MS diagnosis

Biomarkers that are suitable for MS diagnosis must make it possible to differentiate between patients with MS and healthy people or those with other diseases.

### Oligoclonal bands

Oligoclonal bands are bands of immunoglobulins that are seen when patient’s blood serum and CSF are analyzed in parallel. It has been known for some time that oligoclonal bands (OCB) occur in the analysis of CSF (by isoelectric focusing) in MS patients [48]. They are created by immunoglobulin G (IgG) and M (IgM) produced by plasma cells in the CNS [49]. The existence of these bands within the CSF, but not within the serum, is a strong indicator of intrathecal antibody synthesis and, interestingly, is found in nearly all patients with clinically definitive MS. Intrathecal antibodies are mainly produced by plasma cells (terminally differentiated B cells), and hence an involvement of B cells in the pathogenesis of MS has long been suspected [49]. In more than 95% of MS patients, OCB are detectable in the cerebrospinal fluid, but mostly not in serum [50]. However, OCB are not MS specific and can also occur in other inflammatory CNS diseases [51]. If other diagnoses are excluded though, OCB support the diagnosis of MS. They [49] were already introduced in 1983 as a diagnostic criterion in MS and thus represent the first biomarker of this disease [52]. After OCB have meanwhile not been used for diagnosis according to the McDonald criteria, they are now again part of the diagnostic algorithm in the updated version of 2017 [53]. This shift to substitute of a positive CSF result for dissemination in time rather than to substitute for dissemination in space is a practical one, but it reinforces the responsibility of clinical neurologists to request state-of-the-art CSF analyses [54, 55]: Patients with typical clinical presentations, typical lesions, and with alternative diagnoses reasonably ruled out most probably have multiple sclerosis. Demonstrating the presence of OCB will provide supporting evidence of the immune and inflammatory nature of the disease without having to wait for dissemination in time to occur [53, 55, 56]. OCB are thus an established biomarker with significance for MS diagnosis.

### IgG Index

The immunoglobulin (Ig) G index describes the ratio of the CSF/serum quotient of IgG to the CSF/serum quotient of the reference protein albumin [57]. The albumin quotient, albumin in CSF/albumin in serum, is used as a measure of blood-CSF barrier dysfunction in MS [58]. IgG index is used a marker of intrathecal production of immunoglobulins. A value of IgG index > 0.7 is an indicator of an increased intrathecal B cell response and thus indicates the presence of MS [18]. About 70% of MS patients have an increased IgG index [59, 60]. Hence, the sensitivity of this biomarker is albeit lower than that of the OCB [50]. Furthermore, an increased IgG index rarely occurs in MS patients without OCB. Nevertheless, the IgG index is one of the established biomarkers of MS diagnosis and is regularly determined in the course of CSF diagnostics.

### Measles, rubella, varicella-zoster reaction

If antibodies against the neurotrophic viruses, measles virus, rubella virus, and varicella-zoster virus (VZV), are detected in the CSF, this suggests a poly-specific intrathecal B cell response. Therefore, the determination of measles, rubella, varicella-zoster (MRZ) reaction is one of the recommended measures in cases of suspected MS [61, 62]. Brettschneider and colleagues also showed that an MRZ reaction is significantly more frequently detectable in patients with a conversion from clinically isolated syndrome (CIS) to MS than in patients who do not develop clinically definite MS [63]. This finding supports the notion that immunological changes related to B cell activation and intrathecal polyspecific IgG synthesis occur early on in the development of MS [63]. In MS, the polyspecific intrathecal MRZ humoral response seems to show the enhanced B cell-promoting environment.

### Anti-aquaporin-4 antibodies

Aquaporin-4 (AQP-4) is a water channel protein expressed in the CNS by astrocytes which plays a major role in the regulation of water homeostasis in the CNS [64–67]. Antibodies against this protein are detectable in about 75% of patients with neuromyelitis optica spectrum disorder (NMOSD), but not in MS patients [68]. This makes anti-aquaporin-4 antibodies exemplary for biomarkers with a high specificity. It is the first clinically established molecular biomarker that enables differentiation between various inflammatory demyelinating diseases of the CNS. Detection of anti-aquaporin-4 antibodies is usually performed in serum in patients suspected of having NMOSD [69]. Different detection methods are available: indirect immunofluorescence, ELISA, flow cytometry, and cell-based assays [70]. The latter are characterized by particularly high specificity and sensitivity and are therefore recommended for the detection of anti-aquaporin-4 antibodies [71].

### Anti-MOG antibodies

MOG is a myelin protein expressed exclusively on the surface of myelin sheaths and membranes of oligodendrocytes and a potential target molecule for the autoimmune response in demyelinating diseases [72–74]. Unlike initially postulated, anti-MOG antibodies are not suitable for the diagnosis or prognosis of MS, but rather for differential diagnosis. Using state-of-the-art detection methods (cell-based methods), it was shown that anti-MOG antibodies are found in a subgroup of pediatric patients with acute disseminated encephalomyelitis (ADEM), patients with clinical symptoms of NMOSD, and patients with bilateral optic neuritis in particula r[75]. In classical MS, however, high anti-MOG antibody titres are rare, with the frequency of seropositive MS patients being highest in the pediatric patient group. In a study by McLaughlin and colleagues, the prevalence of anti-MOG antibodies was 38.7% in patients with an initial clinical event under 10 years of age, whereas only 4.3% of patients with onset of the disease in adulthood (> 18 years of age) were seropositive [76]. By comparing this clearly distinct cohort to AQP-4+ NMO as well as MS, we propose that MOG+ CNS demyelinating disease represents a distinct novel disease entity [77, 78]. So far, anti-MOG antibodies are not routinely determined biomarkers in clinical practice despite these new findings.

### Antinuclear antibodies

Antinuclear antibodies (ANA) are tissue non-specific autoantibodies against components of the cell nucleus, the concentration of which is determined in the serum [79]. According to the guidelines of the German Neurological Society, the ANA test is an obligatory laboratory test for differential diagnosis [80]. A persistently high titer indicates collagenoses such as systemic lupus erythematosus (SLE) [81]. However, in a recent publication, Becker and colleagues discussed whether a positive ANA test without clinical evidence of connective tissue disease is helpful and concluded that testing without suspicion should be well considered [82]. They also suggested that the antibodies against double-stranded DNA (dsDNA), which are also typical for SLE, should only be determined after a positive ANA test result. In the German guideline, however, the detection of anti-dsDNA antibodies is also one of the obligatory laboratory tests for differential diagnosis [80].

## Molecular biomarkers for MS prognosis

Biomarkers for MS prognosis can provide information on the course of disease activity and indicate conversion to another form of MS, for example from CIS to relapsing-remitting MS (RRMS) or from RRMS to secondary progressive MS (SPMS).

### Oligoclonal bands

The detection of oligoclonal IgG bands in CSF is associated with a conversion from CIS to MS and can therefore be described as a biomarker for MS prognosis. For example, a study of Tintore and colleagues with 1015 patients showed that oligoclonal IgG bands increased the risk of clinically confirmed MS (adjusted hazard ratio 1.3 [1.0–1.8]) and disability accumulation (adjusted hazard ratio 2.0 [1.2–3.6]) independently of other factors [83]. In addition, in an examination of Kuhle and colleagues, oligoclonal IgG bands proved to be the strongest prognostic factor for conversion from CIS to MS, along with the lesion load and the age at the onset of the disease [84]. A recent study also showed a prognostic significance of oligoclonal IgG bands in the conversion of radiologically isolated syndrome (RIS) to CIS [85]. OCB can also result from the production of IgM in the CNS. In some studies of, so far, only one Spanish research group, these oligoclonal IgM bands have been associated with an increased risk of conversion from CIS to MS and with an aggressive course of the disease [86, 87]. However, there are also studies that show no correlation of oligoclonal IgM bands with the MS prognosis [88]. Therefore, the usefulness of oligo IgM as prognostic marker remains to be confirmed by future studies.

### Chitinase-3-like-1

The protein chitinase-3-like-1 is a glycosidase secreted by monocytes, microglia, and activated astrocytes [89]. The physiological role of chitinase-3-like-1 (CHI3L1) in the CNS is unknown; however, its distribution in inflammatory lesions suggests that it might be an important component of the astrocytic response to modulate CNS inflammation CHI3L1 1 [90–92]. It is usually detected in the CSF. Cantó and colleagues showed in a multicentre longitudinal cohort study with 813 participants that the CHI3L1 concentration is an independent risk factor for the conversion from CIS to MS. High CHI3L1 levels were also associated with faster disability progression [93]. Although CHI3L1 is not yet clinically established, it is a promising candidate as a biomarker of MS prognosis and probably treatment response [94].

### Neurofilaments

Neurofilaments (NF) are neuronal cytoskeletal proteins consisting of a light (NFL), an intermediate (NFM), and a heavy (NFH) chain [95]. They determine the diameter of axons and are involved in axonal transport. If axonal or neuronal damage occurs, NF are released and can be detected in the CSF and blood [96]. For detection in blood, an ultra-sensitive technique called single molecule arrays (SIMOA) has been developed only recently, for the first time allowing the detection of NFL in serum [97]. Compared to detection using ELISA or electrochemiluminescence (ECL) based assays, SIMOA is characterized by > 25 times higher analytical sensitivity (SIMOA: 0.62 pg/ml, ECL assay: 15.6 pg/ml, ELISA: 78.0 pg/ml) [98]. NFL are also highly stable and insensitive to the usual storage conditions, which increases the robustness of the detection methods [99]. According to a study by Disanto and colleagues, MS patients have elevated NFL levels compared to the control group, with a strong association of values measured simultaneously in CSF and serum [100]. Serum NFL levels also correlate with MRI activity, degree of disability, and brain atrophy rate [100–102]. Furthermore, NFL is also suitable as a prognostic biomarker for the conversion from CIS to MS [18, 103]. A recent study also showed a prognostic significance of serum NFL in the conversion from RIS to CIS [85].

Overall, the determination of serum NFL concentration, which does not necessarily require a lumbar puncture but can be now measured in the blood, seems to correlate with many clinical and magnetic tomographic characteristics of MS [96, 104, 105]. A future establishment as a prognostic biomarker in clinical practice is therefore conceivable. While NFL measurement in serum is a well-established marker of neuroaxonal damage in MS, there are promising data on astroglial markers in serum as glial fibrillary acid protein (GFAP) [106, 107].

## Molecular biomarkers for monitoring therapy response

Thanks to the progressive elucidation of the MS pathophysiology, a number of disease-modifying therapies with specific mechanisms of action are now available. However, not all patients respond equally to treatment. In order to be able to treat each patient with the individually optimized MS treatment at the right time, it is necessary to know biomarkers for predicting the therapeutic response and monitoring its effectiveness.

### Neutralizing antibodies against interferon-β

Neutralizing antibodies can be formed in response to the administration of mostly protein drugs and prevent its actual mechanism of action. Such antibodies are detected in serum. In interferon therapy (IFN)-β, neutralizing antibodies are produced in up to 40% of patients, depending on the type of IFN. This usually occurs during the first 2 years of treatment [108]. Neutralizing antibodies against IFN-β have been shown to reduce its positive effect on annual relapse rate, disability progression and MRI activity [12]. Therefore, a change of therapy is recommended within 3 to 6 months if two positive test results are obtained [109]. Neutralizing antibodies against IFN-β therefore represent a prognostic biomarker for poor therapy response. An indirect biomarker for the biological activity of IFN-β is the myxovirus resistance protein A (MxA), an antiviral protein selectively induced by IFN-β [12, 110, 111]. In this case, detection takes place by expressing MxA mRNA in blood cells. If neutralizing antibodies against IFN-β with a low to medium titer were detected in a patient, the MxA amount can be determined as additional information. With a low MxA level meaning low IFN-β bioavailability, a change in therapy should be considered [109].

### Neutralizing antibodies against natalizumab

Neutralizing antibodies can also be formed during therapy with natalizumab, the monoclonal antibody against integrin α4β1 and α4β7 on leukocytes. In an average of 6% of patients treated with natalizumab, neutralizing antibodies are detected at least once. In > 90% of cases, these occur during the first three months of treatment [12]. The neutralizing antibodies lower the serum level of natalizumab and, with continuous presence, are associated with a reduced efficacy of the therapy [112, 113]. For example, a study by Vennagoor and colleagues showed an association of high neutralizing antibody titers with the occurrence of episodes and gadolinium-enhancing lesions in MRI [114]. Although there are currently no guidelines for the routine use of neutralizing antibodies against natalizumab as prognostic biomarkers for therapy response, it is recommended that a corresponding test should be performed within 3 to 4 months after the start of therapy (in almost all cases the antibodies are formed within the first 4 to 6 months) and when relapses occur [12, 115, 116]. Since neutralizing antibodies are also associated with the occurrence of infusion-related side effects, they also represent a biomarker for therapeutic side effects [117]. Neutralizing antibodies may be of relevance for other monoclonal antibodies as well such as Ocrelizumab depleting CD20+ B cells or Alemtuzumab depleting CD52+ cells.

### Neurofilament light chain

Biomarkers that show a correlation with disease activity in RRMS patients can provide important indications for therapeutic response. Since the release of NFL is related to the occurrence of axon damage and the NFL concentration correlates with disease activity, the protein could be such a biomarker for therapeutic response [96, 118, 119]. Several studies have already shown an average decrease in the amount of NFL in CSF of MS patients following treatment with natalizumab [120], fingolimod [121], mitoxantrone or rituximab [122], or alemtuzumab [123]. For example, Gunnarsson and colleagues observed a decrease in NFL levels to about the level of healthy controls 6 to 12 months after the start of natalizumab therapy [120]. Treatment with fingolimod also led to a significant decrease in NFL levels in CSF after 12 months according to a study by Kuhle and colleagues, whereas no significant change occurred in the placebo group [121]. A decrease in NFL levels was also observed in serum after treatment with progression-modifying therapies, including natalizumab, fingolimod, and mitoxantrone [124, 125]. In a study by Akgün et al. in alemtuzumab-treated patients using monthly serum NFL (sNFL) assessment, clinical or MRI disease activity was paralleled by an increase of sNFL level (increase up to 20-fold for 3–6 months) [123]. Even patient-reported symptoms that have not been classified as clinical relapse before were accompanied by sNFL increase proposing sNFL assessment to proof a relapse. Usually, sNFL increased about 1 month prior to first clinical symptoms with further increase and recovery over the following 1 to 3 months. Monthly sNFLs presented at higher values in patients with disease activity that required alemtuzumab retreatment compared to responder patients.

### C-X-C motif chemokine-13

The C-X-C motif chemokine-13 (CXCL13) is one of the most potent B cell chemoattractants and is significantly involved in the recruitment of B cells into the CNS in MS. Consequently, increased levels of CXCL13 in the CSF of MS patients could be measured compared to healthy controls. In addition, a correlation of elevated CXCL13 levels with disease activity was shown [126]. In a study by Novakova and colleagues, patients with natalizumab therapy had lower CXCL13 values than patients receiving IFN-β therapy [127]. Another study also observed a reduction in CXCL13 levels after conversion from IFN-β, glatiramer acetate, or teriflunomide to fingolimod [128]. According to these results, CXCL13 could be a suitable biomarker for the efficacy of MS therapies. At present, however, it is not yet used clinically.

## Molecular biomarkers for therapeutic side effects

In addition to clinical response, adverse events are a decisive criterion for the success of a therapy. Molecular biomarkers can be an important tool for predicting and monitoring side effects.

### Anti-varicella zoster virus antibodies

Antibodies against VZV are an established biomarker for side effects of various RRMS therapies. Recently, we have shown that the antibody level is in quite good correlation with the more relevant cellular VZV response which is difficult to quantify [129]. Due to the altered immune response, the risk of herpetic infections is increased with some immunomodulating therapies [130–132]. To avoid VZV reactivation in the course of therapy, the anti-VZV antibody titer should be determined in serum before starting treatment with fingolimod, alemtuzumab, and cladribine in patients without previous chickenpox disease or vaccination [133, 134]. In the case of seronegative status, vaccination should be carried out and the start of therapy should be postponed by 4 to 6 weeks in order to fully establish vaccination protection. Prophylactic administration of antiherpetics is also recommended for all patients who are treated with alemtuzumab [133]. In cladribine therapy, herpes prophylaxis should be considered if the lymphocyte counts drop below 200/μl for the duration of grade 4 lymphopenia [135].

### Anti-John Cunningham virus antibodies

Antibodies against the John Cunningham virus (JCV) are detected in serum or plasma and represent a risk factor for the development of progressive multifocal leukoencephalopathy (PML) during treatment with natalizumab. The risk of PML is also increased by prior immunosuppressive therapy and the duration of natalizumab treatment [136]. Anti-JCV antibody positive patients without prior immunosuppressive therapy are additionally differentiated according to the Anti-JCV antibody index (equivalent to the strength of the ELISA reaction) for PML risk assessment [137]. Accordingly, the risk of developing PML increases significantly in patients with an index value > 1.5. Close monitoring and, if necessary, a treatment switch are appropriate in this case. Thus, anti-JCV antibodies are an established and important biomarker in natalizumab therapy. Our risk estimates calculated from patient-level clinical data allow individualized annual prediction of risk of PML in patients receiving natalizumab for multiple sclerosis, supporting yearly benefit–risk re-evaluation in clinical practice [138]. However, they do not provide absolute certainty in predicting PML and do not allow PML risk assessment in other therapies.

### L-selectin expression

L-selectin (CD62L) is an adhesion molecule on the cell surface of lymphocytes. The proportion of CD62L-expressing CD4^+^ T cells in peripheral mononuclear blood cells is another biomarker candidate for the PML risk in natalizumab therapy [139]. Schwab and colleagues found, for example, a correlation of the CD62L values with the JCV serostatus and the JCV index [140]. In addition, in this study with 17 pre-PML patients and 1410 control patients, a low CD62L proportion increased the risk of developing PML by a factor of 55. However, another study with 21 PML patients treated with natalizumab and 104 control group patients treated with natalizumab showed no correlation between CD62L and PML risk [141]. A further comprehensive validation is necessary in this case in order to clarify the suitability of CD62L as a biomarker for therapeutic side effects.

## Conclusions

Molecular biomarkers enable individual decisions and are an important step on the way to a personalized therapy [3, 142]. An ideal biomarker is characterized by high sensitivity and specificity as well as a simple, cost-effective, reproducible, and non-invasive detection method. At present, the diagnosis and prognosis of MS as well as the monitoring of treatment response and the assessment of the risk of side effects can be facilitated with the help of some established biomarkers. These include oligoclonal bands and the IgG index, anti-AQP-4 antibodies, neutralizing antibodies against IFN-β and natalizumab, as well as anti-JCV and anti-VZV antibodies. In addition, there are promising biomarker candidates such as NFL and CHI3L that need to be validated in further studies. However, long-term studies in large cohorts are necessary to promote the application of biomarker candidates in clinical practice. Despite these initial successes, biomarkers that enable a reliable prediction of the therapy response even before the start of treatment and thus individualized therapy are still lacking. There is therefore still a need to develop and validate new biomarkers in the field of MS.

## Data Availability

N/A.

## Abbreviations

ADEM: Acute disseminated encephalomyelitis
ANA: Antinuclear antibodies
AQP-4: Aquaporin-4
CHI3L1: Chitinase-3-like-1
CIS: Clinically isolated syndrome
CNS: Central nervous system
CSF: Cerebrospinal fluid
CXCL13: C-X-C motif chemokine-13
DNA: Deoxyribonucleic acid
Ds: Double-stranded
ECL: Electrochemiluminescence
ELISA: Enzyme-linked immunosorbent assay
GFAP: Glial fibrillary acid protein
IFN: Interferon
Ig: Immunoglobulin
IL: Interleukin
JCV: John Cunningham virus
MBP: Myelin basic protein
MOG: Myelin oligodendrocyte glycoprotein
MRI: Magnetic resonance imaging
MRZ: Measles rubella varicella zoster
MS: Multiple sclerosis
MxA: Myxovirus resistance protein A
NF: Neurofilament
NFH: Neurofilament heavy
NFL: Neurofilament light
NFM: Neurofilament intermediate
NMOSD: Neuromyelitis optica spectrum disorder
OCB: Oligoclonal bands
PML: Progressive multifocal leukoencephalopathy
RIS: Radiologically isolated syndrome
RNA: Ribonucleic acid
RRMS: Relapsing remitting multiple sclerosis
SIMOA: Single molecule array
SLE: Systemic lupus erythematosus
sNFL: Serum neurofilament light
SPMS: Secondary progressive multiple sclerosis
VZV: Varicella zoster virus
